# Abnormal expression of rno_circRNA_014900 and rno_circRNA_005442 induced by ketamine in the rat hippocampus

**DOI:** 10.1186/s12888-019-2374-2

**Published:** 2020-01-02

**Authors:** Jing Mao, Tianmei Li, Di Fan, Hongli Zhou, Jianguo Feng, Li Liu, Chunxiang Zhang, Xiaobin Wang

**Affiliations:** 1 School of Clinical Medicine, the Affiliated Hospital of Southwest Medical University, Luzhou, Sichuan Province People’s Republic of China; 2 Laboratory of Anesthesiology, the Affiliated Hospital of Southwest Medical University, Luzhou, Sichuan Province People’s Republic of China; 3 Department of Anesthesiology, the Affiliated Hospital of Southwest Medical University, No.25, Taiping Road, Luzhou, Sichuan Province 646000 People’s Republic of China; 4 Department of Biomedical Engineering, School of Medicine, University of Alabama at Birmingham, Birmingham, AL USA

**Keywords:** Rat, Ketamine, CircRNA, Hippocampus

## Abstract

**Background:**

Recent studies have shown that circular RNA (circRNA) is rich in microRNA (miRNA) binding sites. We have previously demonstrated that the antidepressant effect of ketamine is related to the abnormal expression of various miRNAs in the brain. This study determined the expression profile of circRNAs in the hippocampus of rats treated with ketamine.

**Methods:**

The aberrantly expressed circRNAs in rat hippocampus after ketamine injection were analyzed by microarray chip, and we further validated these circRNAs by quantitative reverse-transcription PCR (qRT-PCR). The target genes of the different circRNAs were predicted using bioinformatic analyses, and the functions and signal pathways of these target genes were investigated by Gene Ontology (GO) and Kyoto Encyclopedia of Genes and Genomes (KEGG) pathway analyses.

**Results:**

Microarray analysis showed that five circRNAs were aberrantly expressed in rat hippocampus after ketamine injection (fold change > 2.0, *p* < 0.05). The results from the qRT-PCR showed that one of the circRNAs was significantly increased (rno_circRNA_014900; fold change = 2.37; *p* = 0.03), while one was significantly reduced (rno_circRNA_005442; fold change = 0.37; *p* = 0.01). We discovered a significant enrichment in several GO terms and pathways associated with depression.

**Conclusion:**

Our findings showed the abnormal expression of ketamine-induced hippocampal circRNAs in rats.

## Background

The most commonly used antidepressants today effectively improve symptoms of depression, but require at least 2 weeks to take therapeutic effect. In addition, about two-thirds of depressive patients do not respond to the currently available antidepressants and are prone to relapse [1]. Therefore, the development of new, fast-acting antidepressants is particularly important for patients with depression-induced suicidal tendencies [2].

Recent observations suggest that sub-anesthetic doses of ketamine produce rapid therapeutic effects in depressed patients and in animal models of depression [3–6]. These effects are characterized by a rapid onset of action (within hours) after a single dose, a lasting effect (1 week), as well as an efficacy in patients resistant to traditional antidepressant drugs [7, 8].

In a prior investigation, we found that the antidepressant effect of ketamine was related to the regulation of multiple microRNAs (miRNAs) in neurons [9]. Recent studies have shown that circular RNA (circRNA) is rich in miRNA binding sites. CircRNA plays the role of an miRNA sponge in cells, thereby relieving the inhibitory effect of the miRNAs on their target genes and increasing the expression of these genes. The mechanism by which circRNA inhibits miRNAs to increase the expression of their target genes is called the competitive endogenous RNA (ceRNA) mechanism [10, 11].

Based on the previous reports, the aim of this study was to determine the effect of an antidepressant dose of ketamine on the expression of circRNAs in the hippocampus of rats and examine a possible circRNA-mediated mechanism for ketamine’s action. This work may provide new perspectives on the development of circRNA as a possible drug target.

## Methods

### Animals

These experiments were conducted in male Sprague-Dawley rats (50 days old, 150–200 g), provided by Chengdu Dashuo biological technology Co., Ltd., China (experimental animal production license: SCXK Chengdu 2013–17). These rats were housed 5 per cage in standard cages (42 × 20 × 20 cm) in a room. Animals had access to food and water ad libitum during the experiment. The room was maintained at 25–26 °C with about 65% relative humidity, on a 12-h dark/light cycle (lights on at 7 am). All experiments were performed according to National Institute of Health (NIH) guidelines and approved by the ethics committee of Southwest Medical University (Approval number:20180306038).

### Experimental design and procedure

After 1 week of adaptation, the rats were randomly divided into control and experimental group (12 rats/group). The rats in control group were daily injected with 0.9% saline, whereas the rats in experimental group received ketamine (15 mg/kg). All injections were done intraperitoneally for three consecutive days (the volume of injection was 1 ml/kg). The dose of ketamine used in this study was based on our previous study [9]. On day 4, approximately 24 h after the last ketamine or saline injection, the rats were sacrificed by cervical dislocation, and their hippocampus tissues were dissected out for circRNA microarray analysis (*n* = 3/group) and for qRT-PCR (*n* = 6 /group). The hippocampus of 3 remaining rats per group were spare specimen according to the quality of hippocampus removed from rats.

### CircRNAs analysis from microarray chip

Relevant circRNAs were analyzed according to our previous approach [12]. Hippocampus tissues(*n* = 3/group) were used for microarray assay to determine differentially expressed circRNA between the two groups. The microarray hybridization was performed based on the Arraystar’s standard protocols, including purifying RNA, transcribing into fluorescent cRNA, and hybridizing onto the rat circRNA Arrays (Arraystar). Finally, the hybridized slides were washed, fixed and scanned to images by the Agilent Scanner G2505C. The Agilent Feature Extraction software (version 11.0.1.1) were used to analyze the acquired array images. The raw data were normalized and data analysis was further performed with the R software Limma package (Agilent Technologies). The statistical significance of differentially regulated circRNAs between the two groups was identified through screening fold change ≥2.0, *P* < 0.05 and FDR < 0.05.

### Quantitative real-time PCR validation

Hippocampus tissues(*n* = 6/group) were used for qRT-PCR validation. After RNA isolation, M-MLV reverse transcriptase (Invitrogen, USA) was used for synthesizing cDNA according to the manufacturer’s instructions. Subsequently, we performed qRT-PCR using the ViiA 7 Real-time PCR System (Applied Biosystems, Foster City, CA, USA) in a total reaction volume of 10 μl, including 2 μl cDNA, 5 μl 2 × Master Mix, 0.5 μl PCR Forward Primer (10 μM), 0.5 μl PCR Reverse Primer (10 μM) and 2 μl double distilled water. The protocol was initiated at 95 °C for 10 min, then at 95 °C (10 s), 60 °C (60 s) for a total 40 cycles. β-actin was used as a reference. Results were harvested in three independent wells. For quantitative results, the relative expression level of each circRNA was calculated using 2^−ΔΔCt^ method.

### Competing endogenous RNA analysis of differentially expressed circRNAs

The candidate miRNA binding sites were searched on the sequences of circRNAs and mRNAs, and the circRNA-miRNA-mRNA interaction were found by the overlapping of the same miRNA seed sequence binding site both on the circRNAs and the mRNA. The miRNA-mRNA interactions were predicted by Targetscan (http://www.targetscan.org/), while the miRNA binding sites were predicted by miRcode (http://www.mircode.org/).

### GO and KEGG pathway analysis

Gene Ontology (GO) analysis (http://www.geneontology.org) were conducted to construct meaningful annotation of genes and gene products in the organisms. The ontology includes molecular functions (MFs), biological processes (BPs) and cellular components (CCs). KEGG pathway analysis were also performed to harvest pathway clusters on the molecular interaction and reaction networks in differentially regulated genes. The -log10 (*p*-value) denotes enrichment score representing the significance of GO term enrichment and pathway correlations among differentially expressed genes.

### Statistical analysis

The statistical package for the social sciences (SPSS) 11.0 was selected for statistical analysis. All data were expressed as mean ± SEM. The data from the circRNA microarray and qRT-PCR were analyzed by one-way ANOVA or multi-factorial ANOVA followed by Tukey’s post hoc test. *P* values less than 0.05 with statistically significant differences.

## Results

### The expression profile of circRNAs in the rat hippocampus using microarray analysis and secondary validation from qRT-PCR

The RNA concentration and purity of all samples meted the requirement (larger than 1.8 and an RNA concentration greater than 30 ng) for subsequent microarray detection of the circRNA expression profile.

**Fig. 1:**
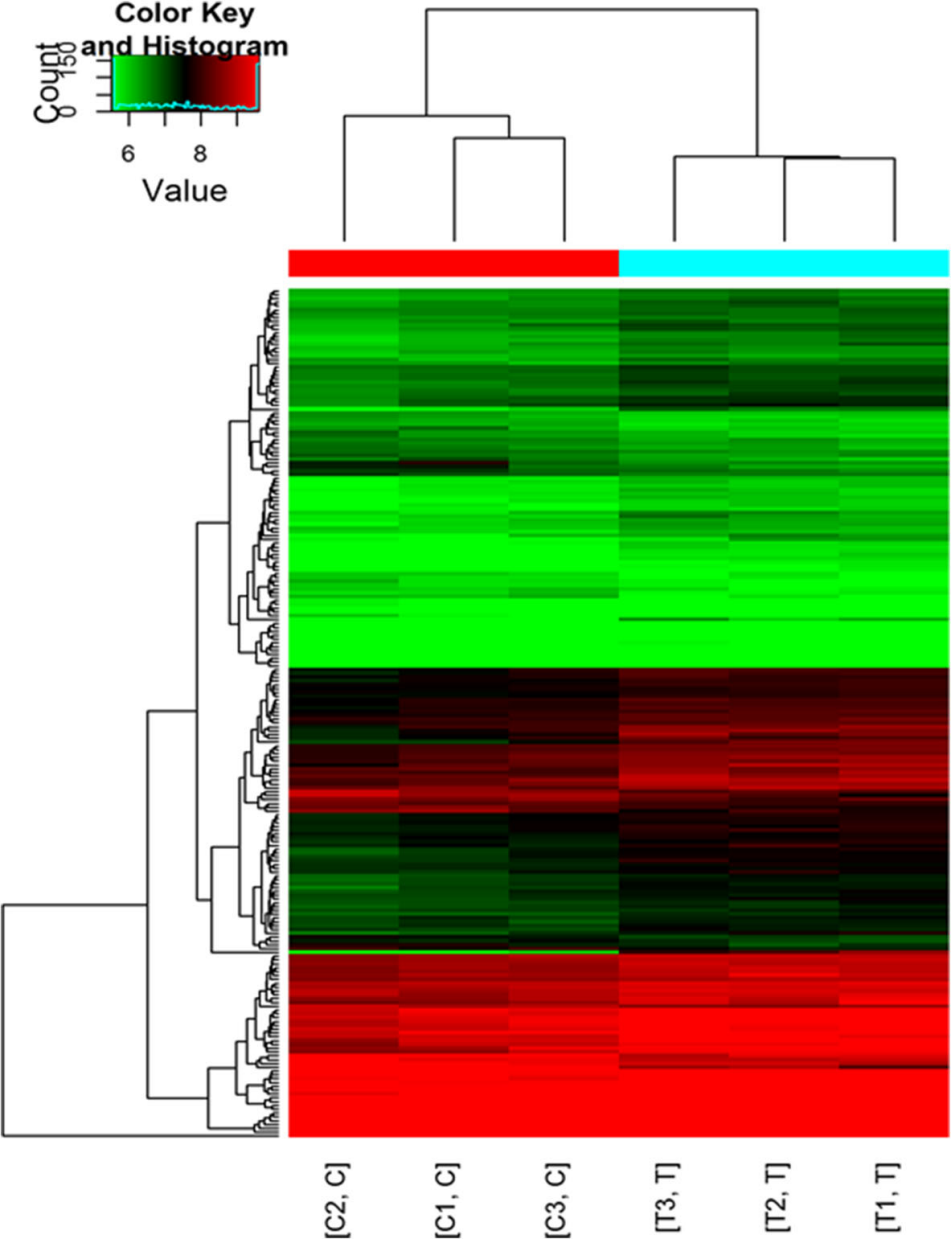
The hierarchical clustering plot of differentially expressed circRNAs (fold change ≥1.5, *p* < 0.05). Red indicates circRNAs with high expression levels, and green represents circRNAs with low expression levels, the color depth (ranging from black to color) indicates different expression intensities. Each row in the figure indicates a different circRNA, and each column indicates a sample (T is the ketamine group and C is the vehicle group). The left side of the figure shows the circRNA clustering tree, whereas the top shows the hippocampal sample clustering tree

As shown in Fig. 1, significantly different circRNAs were selected for hierarchical clustering analysis. The circRNA hierarchical clustering map not only displays circRNA expression, but also exhibits the expression change of a single circRNA from both groups. As shown in Table 1, four circRNAs were upregulated and one was down-regulated in the hippocampus of rats treated with ketamine from circRNA microarray analysis (*p* < 0.05), but only two of the five circRNAs were confirmed to be differentially expressed from qRT-PCR (Table 3, *p* < 0.05). As shown in Table 2, the primers for the five different circRNAs were designed using Primer software 5.0.

**Table 1:** Aberrantly expressed circRNAs in rat hippocampus revealed by microarray analysis (Fold change ≥2.0, *p* < 0.05)

circRNA	Fold Change	Regulation	*P*-value
rno_circRNA_003460	5.98	up	0.001
rno_circRNA_014900	2.28	up	0.003
rno_circRNA_006565	2.11	up	0.010
rno_circRNA_013109	2.17	up	0.012
rno_circRNA_005442	2.01	down	0.012

**Table 2:** A list of primers used for real-time PCR

Gene	Bi-directional primer sequence	Annealing temperature (°C)	Primer length (bp)
β-actin (Reference)	Forward:5′CGAGTACAACCTTCTTGCAGC 3′ Reverse: 5′ ACCCATACCCACCATCACAC 3’	60	202
rno_circRNA_014900	Forward:5′ CTTAGATGACCTGGAGAAGACCT 3′ Reverse: 5′ TGACTTGGTGCTGTTGACTTTAG 3’	60	124
rno_circRNA_013109	Forward:5′ ATTATAGAGCTAATTACAACTTCCG 3′ Reverse:5′ TTATCTGAAGCATGTTAAGACAATA 3’	60	105
rno_circRNA_006565	Forward:5′ CGACTTCAAAAGAGTTGTGGATT 3′ Reverse: 5′ TTCTCCTCGTGAGCTTTTTTCTC 3’	60	54
rno_circRNA_005442	Forward:5′ ACCCCATGAGAAAGACCAGGTC 3′ Reverse:5′ CTGCTCTCTTCAAGTGAAAGACATC 3′	60	60
rno_circRNA_003460	Forward:5′ CGCTAAGCATTTCTTTGGAA 3′ Reverse: 5′ GTAGTGGGTGTAGGGAGGAGA 3′	60	76

### Predicted miRNAs sponged by the two circRNAs and their corresponding target genes

As shown in Table 3, the two circRNAs 014900 and 005442 collectively sponged ten miRNAs, namely, rno-miR-466b-5p, rno-miR-6332, rno-miR-6321, rno-miR-193a-5p, rno-miR-1224, rno-miR-323-5p, rno-miR-107-5p, rno-miR-135b-5p, rno-miR-135a-5p, and rno-miR-344b-5p. Each of these miRNAs has target genes that they endogenously regulate, as shown in Table 4, the two circRNAs could indirectly regulate numerous target genes by their endogenous competition mechanism.

**Table 3:** qRT-PCR-confirmed expression of circRNAs in rat hippocampus and the predicted target miRNAs

circRNA	Fold Change	Regulation	*P*-value	Predicted target miRNAs
rno_circRNA_014900	2.37	up	0.029	rno-miR-466b-5p rno-miR-6332 rno-miR-6321 rno-miR-193a-5p rno-miR-1224
rno_circRNA_005442	2.72	down	0.010	rno-miR-323-5p rno-miR-107-5p rno-miR-135a-5p rno-miR-135b-5p rno-miR-344b-5p

**Table 4:** Target genes regulated indirectly by the two differentially expressed circRNAs through miRNAs

circRNA	Sponged miRNAs	Gene Symbol	Gene Description
rno_circRNA_014900	rno-miR-193a-5p rno-miR-1224	Nova1	neuro-oncological ventral antigen 1
	rno-miR-193a-5p rno-miR-6332	Clasp1	cytoplasmic linker associated protein 1
	miR-193a-5p rno-miR-466b-5p	Rgs4, Mixl1	regulator of G-protein signaling 4; Mix paired-like homeobox 1
	rno-miR-466b-5p rno-miR-6332	Usp37, RGD1566029, Zfp91, Zmiz1, Pbx1	ubiquitin specific peptidase 37; similar to mKIAA1644 protein; zinc finger protein 91; zinc finger, MIZ-type containing 1; pre-B-cell leukemia homeobox 1
	rno-miR-466b-5p rno-miR-1224	Calcoco1	calcium binding and coiled coil domain 1
	rno-miR-466b-5p rno-miR-6321	Brwd3, Nfat5, Cnot7, Camta1	bromodomain and WD repeat domain containing 3; nuclear factor of activated T-cells 5, tonicity-responsive; CCR4-NOT transcription complex, subunit 7; calmodulin binding transcription activator 1
	rno-miR-6332 rno-miR-1224	Prpf4b, Hgs, Gtdc1, Dgkk	pre-mRNA processing factor 4B;hepatocyte growth factor-regulated tyrosine kinase substrate; glycosyltransferase-like domain containing 1;diacylglycerol kinase kappa
	rno-miR-6332 rno-miR-6321	RGD1562037, Usp24,Ssbp2	similar to OTTHUMP00000046255; ubiquitin specific peptidase 24; single-stranded DNA binding protein 2
	rno-miR-6332 rno-miR-6321 rno-miR-466b-5p	Ssbp2, RGD1562037	singe-stranded DNA binding protein 2; similar to OTTHUMP00000046255
	rno-miR-6332 rno-miR-466b-5p rno-miR-1224	Zfp91	zinc finger protein 91
rno_circRNA_005442	rno-miR-107-5p rno-miR-323-5p	Eps8,Rhot1	epidermal growth factor receptor pathway substrate 8, ras homolog gene family, member T1
	rno-miR-107-5p rno-miR-344b-5p	Hn1	hematological and neurological expressed 1
	rno-miR-107-5p rno-miR-135b-5p	Ptk2,Tiam1	protein tyrosine kinase 2;T-cell lymphoma invasion and metastasis 1
	rno-miR-107-5p rno-miR-135a(b)-5p	Slc8a1,Man2a1,Tnpo1,Rgl1	solute carrier family 8 (sodium/calcium exchanger), member 1; mannosidase, alpha, class 2A, member 1;transportin 1; ral guanine nucleotide dissociation stimulator,-like 1
	rno-miR-323-5p rno-miR-344b-5p	Tomm6	translocase of outer mitochondrial membrane 6 homolog (yeast)
	rno-miR-323-5p miR-135a(b)-5p	Shisa7	shisa family member 7
	rno-miR-344b-5p miR-135a(b)-5p	Arel1	apoptosis resistant E3 ubiquitin protein ligase 1
	rno-miR-323-5p rno-miR-107-5p rno-miR-135a(b)-5p	Rhot1	ras homolog gene family, member T1

### GO analysis

**Fig. 2:**
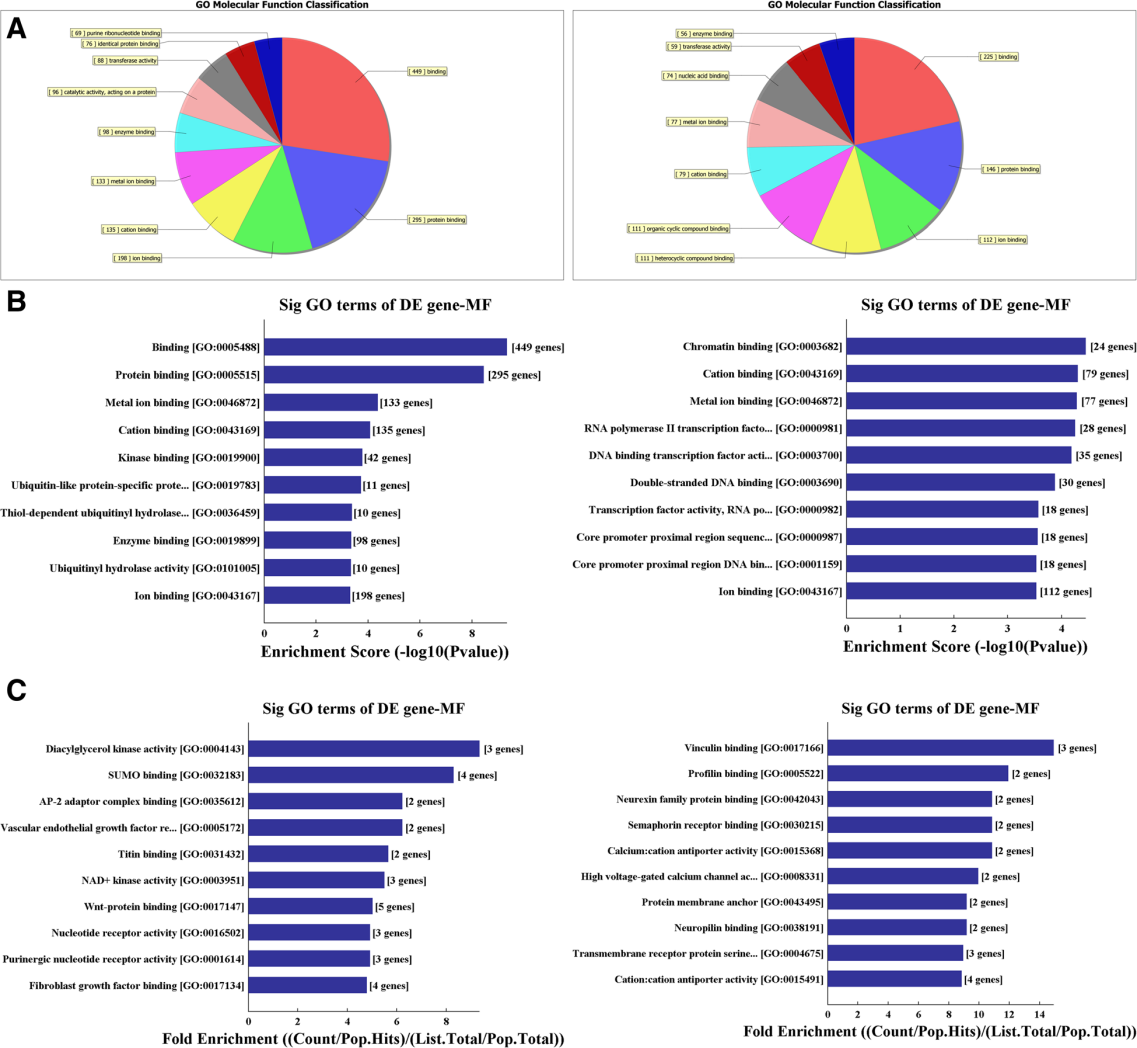
The molecular functions (MFs) of neurons regulated by the target genes of the differentially expressed circRNAs (*p* < 0.05, left: rno_circRNA_014900; right: rno_circRNA_005442). **a** classifies the notable MFs and **b** shows these same MFs ordered by their GO analysis enrichment scores. **c** shows the notable activities of neurons that may be regulated by target genes predicted using fold enrichment

As shown in Fig. 2, the molecular functions (MFs) of neurons that may be regulated by the target genes of the differentially expressed circRNAs (*p* < 0.05, left: rno_circRNA_014900; right: rno_circRNA_005442). The classification of notable MFs was shown in Fig. 2a and b shows the order of these MFs by their GO analysis enrichment scores. Figure 2c shows the notable activities of neurons using fold enrichment. The detailed list of the genes identified as regulated by circRNA was shown in Additional files 1 and 2.

**Fig. 3:**
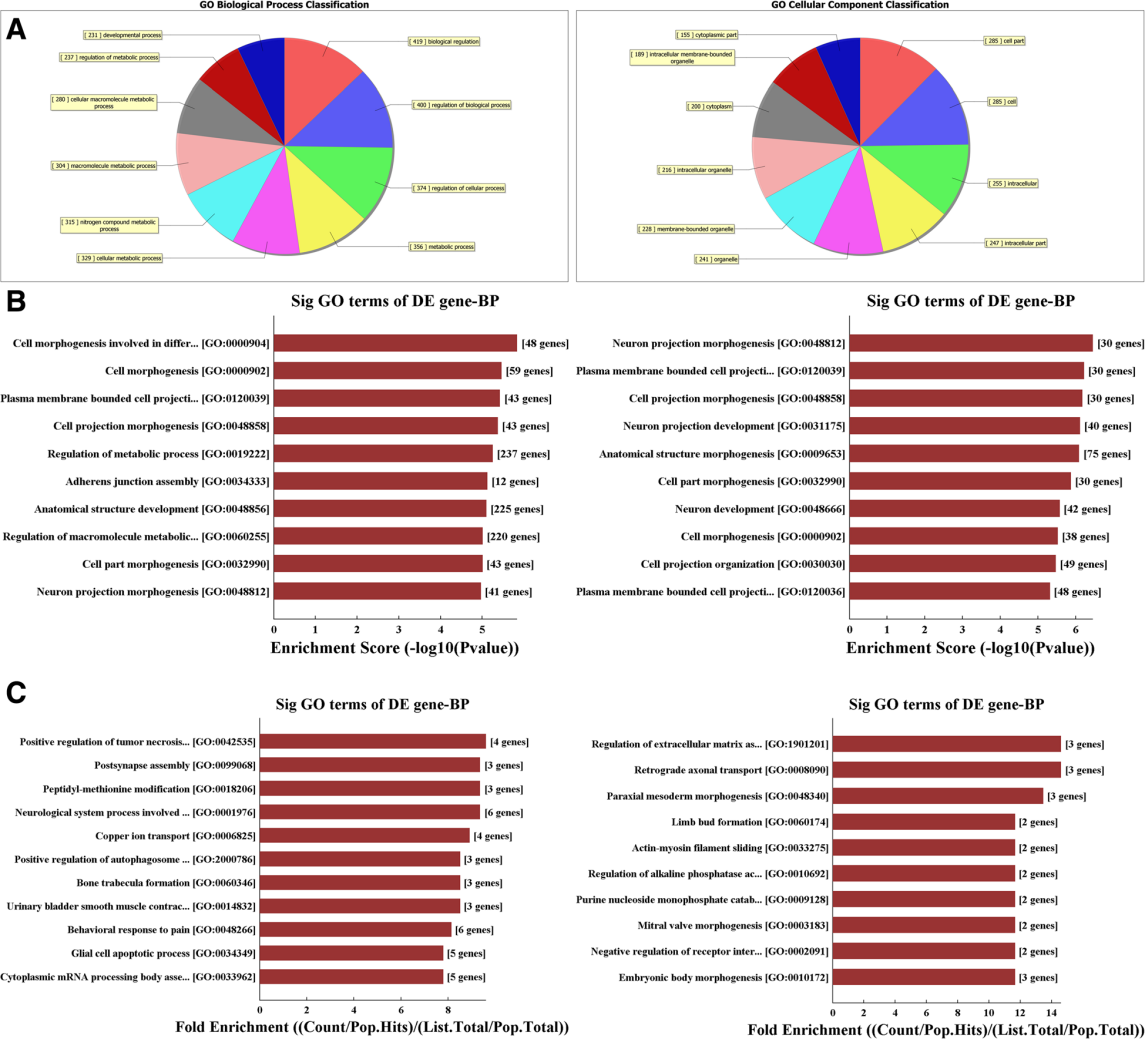
The biological processes (BPs) in neurons regulated by target genes of the two circRNAs (*p* < 0.05, left: rno_circRNA_014900; right: rno_circRNA_005442). **a** classifies the predicted BPs and **b** shows the notable BPs predicted by enrichment scores. **c** shows the notable BPs of neurons that may be regulated by target genes predicted using fold enrichment

As shown in Fig. 3, the biological processes (BPs) in neurons that may be regulated by target genes of the two circRNAs (*p* < 0.05, left: rno_circRNA_014900; right: rno_circRNA_005442). The classification of notable BPs was shown in Fig. 3a and b shows the different BPs predicted by enrichment scores. Figure 3c shows the different BPs of neurons predicted using fold enrichment. The detailed list of the genes identified as regulated by circRNA was shown in Additional files 3 and 4.

**Fig. 4:**
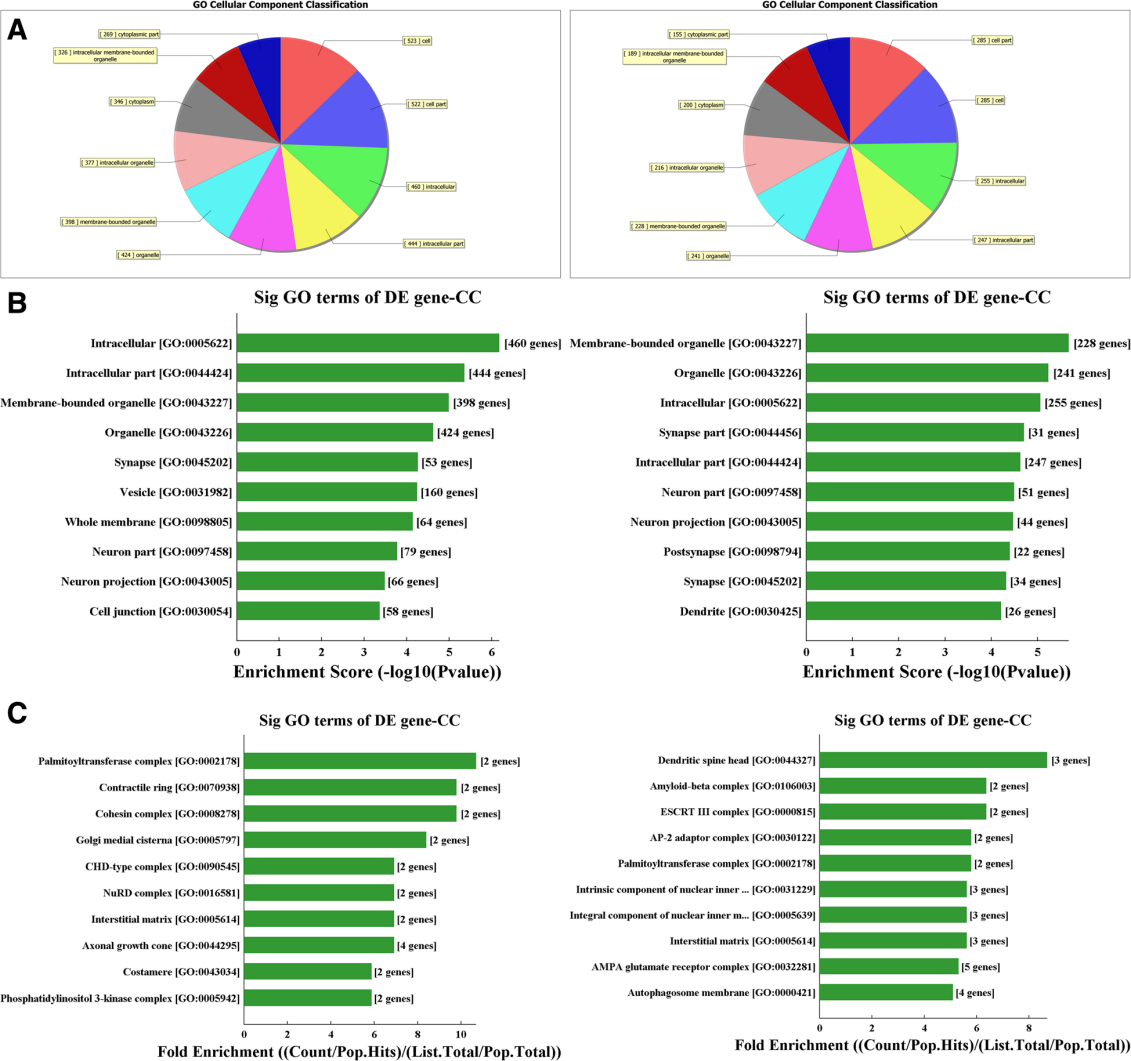
The cellular components (CCs) that may be regulated by target genes of the two differentially expressed circRNAs (*p* < 0.05, left: rno_circRNA_014900; right: rno_circRNA_005442). **a** classifies the predicted CCs and **b** shows the notable CCs predicted by enrichment scores. **c** shows the notable CCs of neurons that may be regulated by target genes predicted using fold enrichment

As shown in Fig. 4, the cellular components (CCs) that may be regulated by target genes of the two differentially expressed circRNAs (*p* < 0.05, left: rno_circRNA_014900; right: rno_circRNA_005442). The classification of notable CCs was shown in Fig. 4a and b shows the different CCs predicted by enrichment scores. Figure 4c shows the different CCs of neurons using fold enrichment. The detailed list of the genes identified as regulated by circRNA was shown in Additional files 5 and 6.

### KEGG pathway analysis

Tables 5 and 6 shows the signaling pathways that may be regulated by target genes of the differentially expressed rno_circRNA_014900 and rno_circRNA_005442 (*p* < 0.05). The identified pathways were involved in the regulation of central nervous system functions, such as Wnt signaling, long-term depression, PI3K-Akt signaling, dopaminergic synapse activity, mTOR signaling, p53 signaling, apoptosis, TGF-beta signaling, axon guidance, hippo signaling, and MAPK signaling.

**Table 5:** The signaling pathways that may be regulated by targeted genes of the differentially expressed rno_circRNA_014900(*p* < 0.05)

PathwayID	Definition	Fisher-Pvalue	Enrichment_Score
rno04510	Focal adhesion - *Rattus norvegicus* (rat)	4.05765E-06	5.391726
rno05205	Proteoglycans in cancer - Rattus norvegicus (rat)	0.000263739	3.578826
rno05200	Pathways in cancer - Rattus norvegicus (rat)	0.000839484	3.075988
rno04216	Ferroptosis - Rattus norvegicus (rat)	0.000947662	3.023347
rno04015	Rap1 signaling pathway - Rattus norvegicus (rat)	0.001242467	2.905715
rno00564	Glycerophospholipid metabolism - Rattus norvegicus (rat)	0.001767738	2.752582
rno04310	Wnt signaling pathway - Rattus norvegicus (rat)	0.002533546	2.596271
rno05165	Human papillomavirus infection - Rattus norvegicus (rat)	0.003213976	2.492957
rno04140	Autophagy - animal - Rattus norvegicus (rat)	0.00464105	2.333384
rno05222	Small cell lung cancer - Rattus norvegicus (rat)	0.005031787	2.298278
rno04070	Phosphatidylinositol signaling system - Rattus norvegicus (rat)	0.006461245	2.189684
rno00561	Glycerolipid metabolism - Rattus norvegicus (rat)	0.00791785	2.101393
rno04213	Longevity regulating pathway - multiple species - Rattus norvegicus (rat)	0.00791785	2.101393
rno04730	Long-term depression - Rattus norvegicus (rat)	0.00791785	2.101393
rno04151	PI3K-Akt signaling pathway - Rattus norvegicus (rat)	0.00874211	2.058384
rno04512	ECM-receptor interaction - Rattus norvegicus (rat)	0.009516369	2.021529
rno04137	Mitophagy - animal - Rattus norvegicus (rat)	0.009920558	2.003464
rno04728	Dopaminergic synapse - Rattus norvegicus (rat)	0.01248382	1.903653
rno04211	Longevity regulating pathway - Rattus norvegicus (rat)	0.01286284	1.890663
rno04136	Autophagy - other - Rattus norvegicus (rat)	0.01346501	1.870793
rno04150	mTOR signaling pathway - Rattus norvegicus (rat)	0.01358861	1.866825
rno04115	p53 signaling pathway - Rattus norvegicus (rat)	0.01402018	1.853246
rno04215	Apoptosis - multiple species - Rattus norvegicus (rat)	0.01492946	1.825956
rno05214	Glioma - Rattus norvegicus (rat)	0.01496318	1.824976
rno04270	Vascular smooth muscle contraction - Rattus norvegicus (rat)	0.02428063	1.61474
rno01521	EGFR tyrosine kinase inhibitor resistance - Rattus norvegicus (rat)	0.02549639	1.593521
rno05219	Bladder cancer - Rattus norvegicus (rat)	0.02580699	1.588263
rno05224	Breast cancer - Rattus norvegicus (rat)	0.02850884	1.54502
rno05166	HTLV-I infection - Rattus norvegicus (rat)	0.02925304	1.533829
rno04066	HIF-1 signaling pathway - Rattus norvegicus (rat)	0.03188594	1.496401
rno04068	FoxO signaling pathway - Rattus norvegicus (rat)	0.03490339	1.457132
rno04144	Endocytosis - Rattus norvegicus (rat)	0.03599259	1.443787
rno04720	Long-term potentiation - Rattus norvegicus (rat)	0.03856442	1.413813
rno04978	Mineral absorption - Rattus norvegicus (rat)	0.04045532	1.393024
rno04725	Cholinergic synapse - Rattus norvegicus (rat)	0.04272807	1.369287
rno05030	Cocaine addiction - Rattus norvegicus (rat)	0.04327309	1.363782
rno04010	MAPK signaling pathway - Rattus norvegicus (rat)	0.04346333	1.361877
rno04120	Ubiquitin mediated proteolysis - Rattus norvegicus (rat)	0.04667443	1.330921
rno00062	Fatty acid elongation - Rattus norvegicus (rat)	0.04768946	1.321578
rno05211	Renal cell carcinoma - Rattus norvegicus (rat)	0.04775832	1.320951
rno01522	Endocrine resistance - Rattus norvegicus (rat)	0.04798442	1.3189

**Table 6:** The signaling pathways that may be regulated by targeted genes of the differentially expressed rno_circRNA_005442 (*p* < 0.05)

PathwayID	Definition	Fisher-Pvalue	Enrichment_Score
rno04350	TGF-beta signaling pathway - Rattus norvegicus (rat)	0.002056745	2.68682
rno04960	Aldosterone-regulated sodium reabsorption - Rattus norvegicus (rat)	0.002806053	2.551904
rno05231	Choline metabolism in cancer - Rattus norvegicus (rat)	0.0039363	2.404912
rno05412	Arrhythmogenic right ventricular cardiomyopathy (ARVC) - Rattus norvegicus (rat)	0.005000479	2.300988
rno04550	Signaling pathways regulating pluripotency of stem cells - Rattus norvegicus (rat)	0.005251424	2.279723
rno04218	Cellular senescence - Rattus norvegicus (rat)	0.005747859	2.240494
rno04360	Axon guidance - Rattus norvegicus (rat)	0.005747859	2.240494
rno05225	Hepatocellular carcinoma - Rattus norvegicus (rat)	0.005747859	2.240494
rno04144	Endocytosis - Rattus norvegicus (rat)	0.006773032	2.169217
rno00510	N-Glycan biosynthesis - Rattus norvegicus (rat)	0.007410522	2.130151
rno04390	Hippo signaling pathway - Rattus norvegicus (rat)	0.009694587	2.013471
rno04068	FoxO signaling pathway - Rattus norvegicus (rat)	0.01587784	1.799209
rno04371	Apelin signaling pathway - Rattus norvegicus (rat)	0.02058289	1.686494
rno04520	Adherens junction - Rattus norvegicus (rat)	0.02501545	1.601792
rno05210	Colorectal cancer - Rattus norvegicus (rat)	0.02501545	1.601792
rno04973	Carbohydrate digestion and absorption - Rattus norvegicus (rat)	0.02580168	1.588352
rno04010	MAPK signaling pathway - Rattus norvegicus (rat)	0.03810185	1.419054
rno05410	Hypertrophic cardiomyopathy (HCM) - Rattus norvegicus (rat)	0.04204027	1.376335
rno05414	Dilated cardiomyopathy (DCM) - Rattus norvegicus (rat)	0.04832562	1.315823

## Discussion

There are about 340 million patients suffering from depression around the world, and it is estimated that up to 1 million people die by depression-induced suicide every year. Therefore, depression has become a global public health problem [13]. The major clinical drawback of currently available antidepressants is their slow onset (14 days on average) and poor effects [1]. Therefore, the development of rapid-onset antidepressants is particularly important for patients suffering from major depressive disorder with suicidal tendency. The antidepressant effect of ketamine initiates quickly and is also effective in treating refractory depression [7, 8]. However, the psychosocial side effects induced by ketamine have restricted its use in the treatment of depression [14]. An in-depth understanding of the mechanism of ketamine’s rapid antidepressant effect can provide new targets for similar antidepressants.

Recent many researches have shown that many transcriptional products come from the non-coding RNA (ncRNA), including the microRNA (miRNA), long non-coding RNA (lncRNA), and circular RNA (circRNA), these ncRNAs can regulate gene expression at the DNA level, pre-transcriptional level, transcriptional level, post-transcriptional level, translational level, and post-translational level [15]. Previous study from our group found that ketamine induced abnormal expression of miRNAs in the brain, and subsequent target gene and functional analysis revealed that this abnormal expression of miRNAs was closely related to the antidepressant effect of ketamine, but the therapeutic effect of antidepressants focusing on a single mechanism is poor due to the complex mechanism of major depressive disorder [9]. Therefore, we believe that a single miRNA plays relatively weak role in the regulation of a specific target gene. It was hypothesized that a substance in the body that could gather several miRNAs regulated the same specific target gene, then the regulatory action on the specific target gene would be obviously increased. It was interesting that circRNAs, a novel type of non-coding RNAs [15], were perceived as a rare curiosity to having a central regulatory role in RNA metabolism, and accumulating evidences suggested that circRNAs could function as miRNA sponges (the ceRNA mechanism), therefore, circRNA could relieve the inhibitory effect of miRNA on its target gene and increase the expression of the target gene [10–12].

Our results showed that the expression of rno_circRNA_014900 was significantly increased by ketamine, while the expression of rno_circRNA_005442 was obviously decreased. Since these two circRNAs were not investigated in previous studies on depression, we further investigated their regulated target genes and signaling pathways. Competing endogenous RNA analysis found that the rno_circRNA_014900 could sponge rno-miR-466b-5p, rno-miR-6332, rno-miR-6321, rno-miR-193a-5p and rno-miR-1224, whereas the rno_circRNA_005442 had the binding sites in rno-miR-323-5p, rno-miR-107-5p, rno-miR-135a-5p, rno-miR-135b-5p and rno-miR-344b-5p. In a prior investigation, we found that miR-206 was a critical novel gene for the expression of BDNF (brain-derived neurotrophic factor) induced by ketamine [9], our experimental results were expected to be that the circRNA identified in this study would target the miRNA-206, but structural prediction analysis showed that the miRNAs sponged by rno_circRNA_014900 and rno_circRNA_005442 did not include miRNA-206, we thought rno_circRNA_014900 and rno_circRNA_005442 did not regulate the expression of miRNA-206, how ketamine affects the expression of miRNA-206 needs to be further explored in future research. Further prediction analysis revealed that rno_circRNA_014900 and rno_circRNA_005442 may inhibit up to four miRNAs closely related to depression. For example, the predicted target genes related to depression include NOVA1 (regulated by miR-193-5p and miR-1224) [16], Rgs4 (regulated by miR-193-5p and miR-466b-5p) [17–20], zinc-finger protein (regulated by miR-6332, miR-466b-5p, and miR-1224) [21], PBX1 (regulated by miR-466b-5p and miR-6332) [22], SLC8A1 (regulated by miR-107-5p, miR-135a(b)-5p, and miR-135b-5p) [23], PTK2 (regulated by miR-107-5p and miR-135b-5p) [24], and Tiam1 (regulated by miR-107-5p and miR-135b-5p) [25]. Therefore, we believed rno_circRNA_014900 or rno_circRNA_005442 could relieve the inhibitory effect of miRNA on its target gene and increase the expression of the target gene, the phenomenon needs to be further confirmed in future studies.

The results from GO analysis found that the the target genes of the two circRNAs regulated many molecular functions (including protein phosphatase binding, SUMO binding, Wnt-protein binding, etc.), biological processes (including adherens junction assembly, neuron projection morphogenesis, neurological processes, etc.), and cellular components (including dendritic spines, AMPA-glutamate receptor binding, neuronal cell body, etc.). The results from KEGG pathway prediction showed that the signaling pathways regulated by target genes of the two circRNAs included Wnt signaling, long-term depression, PI3K-Akt signaling, dopaminergic synapses, mTOR signaling, p53 signaling, apoptosis, MAPK signaling, TGF-beta signaling, axon guidance, Hippo signaling, etc.. These signaling pathways may be involved in the occurrence and development of depression, because some researches found that the Wnt signaling pathway played important roles in the depression-like behaviors [26–28], the PI3K-Akt signaling pathway was related to the rapid antidepressant-like effects of some drugs [29–33].

As we analyzed in other study about circRNAs [12], novel therapies should include multiple genes and pathways due to the complex mechanisms of major depression disorder. Because many pathophysiological processes of stress-related depression were regulated by several miRNAs, therefore these miRNAs may be commom targets of antidepressant therapies. It was not difficult to understand that the increase in relevant circRNAs expression would enhance the translation of their target genes due to miRNA sponges, a down-expression in relevant circRNAs would result in the obvious silencing of downstream target genes. Therefore, in the future, artificial circRNA drugs will be developed to restore normal transcriptional regulation.

## Conclusions

In summary, we found that ketamine treatment resulted in the abnormal expression of the two circRNAs in the hippocampus of rats, and these two circRNAs may be associated with stress-related depression disorders. CircRNAs should remain the focus of researches investigating antidepressant targets because they have considerable potential in the clinical treatment of stress-related depression. As an invaluable topic for future biomedical studies, we plan to screen for specific circRNAs in the context of depression and to examine their potential value in the diagnosis and treatment of this debilitating disorder.

## Supplementary information

**Additional file 1.** MF results for circRNA01490. The detailed list of the genes identified as regulated by the circRNA 01490, MF: molecular function.

**Additional file 2.** MF results for circRNA005442. The detailed list of the genes identified as regulated by the circRNA005442, MF: molecular function.

**Additional file 3.** BP results for circRNA01490. The detailed list of the genes identified as regulated by the circRNA 01490, BP: biological process.

**Additional file 4.** BP results for circRNA005442. The detailed list of the genes identified as regulated by the circRNA005442, BP: biological process.

**Additional file 5.** CC results for circRNA01490. The detailed list of the genes identified as regulated by the circRNA 01490, CC: cellular component.

**Additional file 6.** CC results for circRNA005442. The detailed list of the genes identified as regulated by the circRNA005442, CC: cellular component.

## Data Availability

The data sets used and/or analyzed during the current study are available from the corresponding author on reasonable request.

## Abbreviations

BPs: Biological processes
CCs: Cellular components
ceRNA: Competitive endogenous RNA
circRNA: Circular RNA
GO: Gene Ontology
i.p.: Intraperitoneal
KEGG: Kyoto Encyclopedia of Genes and Genomes
lncRNA: Long non-coding RNA
MFs: Molecular functions
miRNA: MicroRNA
ncRNA: Non-coding RNA
qRT-PCR: Quantitative reverse-transcription PCR
SEM: Standard error of the mean
